# Elevated CO_2_ Suppresses the Vanadium Stress in Wheat Plants under the Future Climate CO_2_

**DOI:** 10.3390/plants12071535

**Published:** 2023-04-02

**Authors:** Emad A. Alsherif, Hamada AbdElgawad

**Affiliations:** 1 Biology Department, College of Science and Arts at Khulis, University of Jeddah, Jeddah 21959, Saudi Arabia; 2 Integrated Molecular Plant Physiology Research, Department of Biology, University of Antwerp, 2180 Antwerp, Belgium

**Keywords:** vanadium, climate change, antioxidant, tocopherol, phytochelatins

## Abstract

Increases in atmospheric CO_2_ is known to promote plant growth under heavy metals stress conditions. However, vanadium (V) stress mitigating the impact of eCO_2_ as well as the physiological and biochemical bases of this stress mitigation have not been well studied. To this end, this study investigated the growth, photosynthetic parameters, oxidative damages antioxidants, and antioxidants enzymes in wheat plants grown under ambient (420 PPM) and high eCO_2_ (720 ppm) levels. Exposing wheat plants to higher V increased its accumulation in plants which consequentially inhibited plant growth and induced oxidative damage. An increase in antioxidant and detoxification defense systems was observed but it was not enough to reduce V stress toxicity. On the other hand, wheat growth was improved as a result of reduced V uptake and toxicity on photosynthesis under eCO_2_. To reduce V uptake, wheat accumulated citric acid, and oxalic acid in soil preferentially under both treatments but to more extend under V and eCO_2_. Additionally, improved photosynthesis induced high carbon availability that was directed to produce chelating proteins (metallothioneins, phytochelatin) and antioxidants (phenolics, flavonoids, total antioxidant capacity). This study advances our knowledge of the processes behind the variations in the physiological and biochemical responses of the wheat crop under V and eCO_2_ conditions.

## 1. Introduction

Future climate change and soil degradation from heavy metals are two big problems that should be taken very seriously [1,2,3]. Globally, atmospheric carbon dioxide (eCO_2_) levels are expected to increase due to the burning of fossil fuels and changes in land use [4]. These events occur even now all across the world [5]. Future crop production profitability and sustainable and equitable food security depend on a better understanding of climate change and shifts in greenhouse gas concentration [6]. Increased CO_2_ is frequently used in greenhouse production of various decorative and agricultural crops to boost yields [7]. According to the IPCC [8], the effect of doubling CO_2_ on a variety of plants ranges from a 10% jump to a nearly 300% rise in biomass. According to an analysis of hundreds of research studies, food and flower crops responded to a doubling of [CO_2_] by increasing their output by an average of 30% (depending on the availability of nutrients and water) [7,9].

Changes in growth, photosynthesis, metabolite partitioning and translocation, photosynthetic enzymes, respiration rate, leaf area index, stomatal conductance, transpiration rate, biomass output, and water usage efficiency are among the potential consequences of high CO_2_ on plants [10]. Under controlled settings, changes in photosynthesis, biomass production, and nutrient relationships were generally examined at the physiological level while the effects of eCO_2_ were explored [11,12]. To achieve this goal, raising atmospheric CO_2_ levels may help plants tolerate HM toxicity in the environment [13]. According to earlier research, in this situation, eCO_2_ lessened the effects of environmental stressors on plant metabolism and growth [14]. For instance, eCO_2_ reduced the negative effects of various HM on the metabolism and growth of plants [15,16,17]. Due to eCO_2_, adding extra resources (carbon) physiologically causes the metabolism of the plant to be redirected toward the production of several stress-related metabolites. In this way, increasing photosynthetic C absorption via eCO_2_ increased the buildup and breakdown of non-structural carbohydrates via dark respiration [18]. As a result, metabolic energy is supplied for the synthesis of a variety of metabolites, including osmoprotectants and antioxidants [19]. Despite having a significant impact on the climate, eCO_2_ levels have been proven to enhance plant growth and output by promoting the photosynthetic uptake of C and lowering photorespiration, particularly in C_3_ plants [11,20,21]. Also, Zinta et al. [21,22] found that eCO_2_ makes plants stronger in bad conditions by making them use water more efficiently and speeding up the metabolism of their antioxidant defense. The rapid industrial expansion has significantly increased the release of contaminants, such as heavy metals (HMs), in many ecosystems, in addition to the environmental effects of global warming [23,24]. As a result, the interaction between HMs and global change would have an influence on agriculture, influencing crop development and growth, which would have an immediate impact on production and food safety [25,26]. Climate change is crucial since several environmental conditions influence the mobilization of HMs in crops [27,28,29,30]. According to Shah et al. [30], HM pollution of soil results in oxidative stress in plants by disrupting enzyme function and substituting necessary metals and nutrients, which has an adverse effect on crop quality [31].

Vanadium (V) is the fifth most common transition element and ranks 22nd among all of the elements found in the earth’s crust. It is typically found in limestones along with other components such as iron oxide and organic waste [32]. Vanadium is one of the non-essential elements for plants that can help with growth and yield, but when its concentration inside plant tissues goes above a specific threshold, it is bad for plant metabolism and eventually slows down development and productivity [32]. Since vanadate and phosphate have similar structures, vanadium oxides and phosphorus can compete for absorption by the roots. As a result of this, vanadium is bad for a plant’s health [33]. Many studies have examined the impact of climate change on crops due to a rise in CO_2_ primarily in terms of quality and productivity [33,34,35]. No studies so far have examined the impact of eCO_2_ in V-polluted soils. The aim of this study was to investigate, for the first time, V stress mitigating impact of eCO_2_ as well as the physiological and biochemical bases of this protective effect.

## 2. Results

### 2.1. Growth and V Accumulation

**Figure 1:**
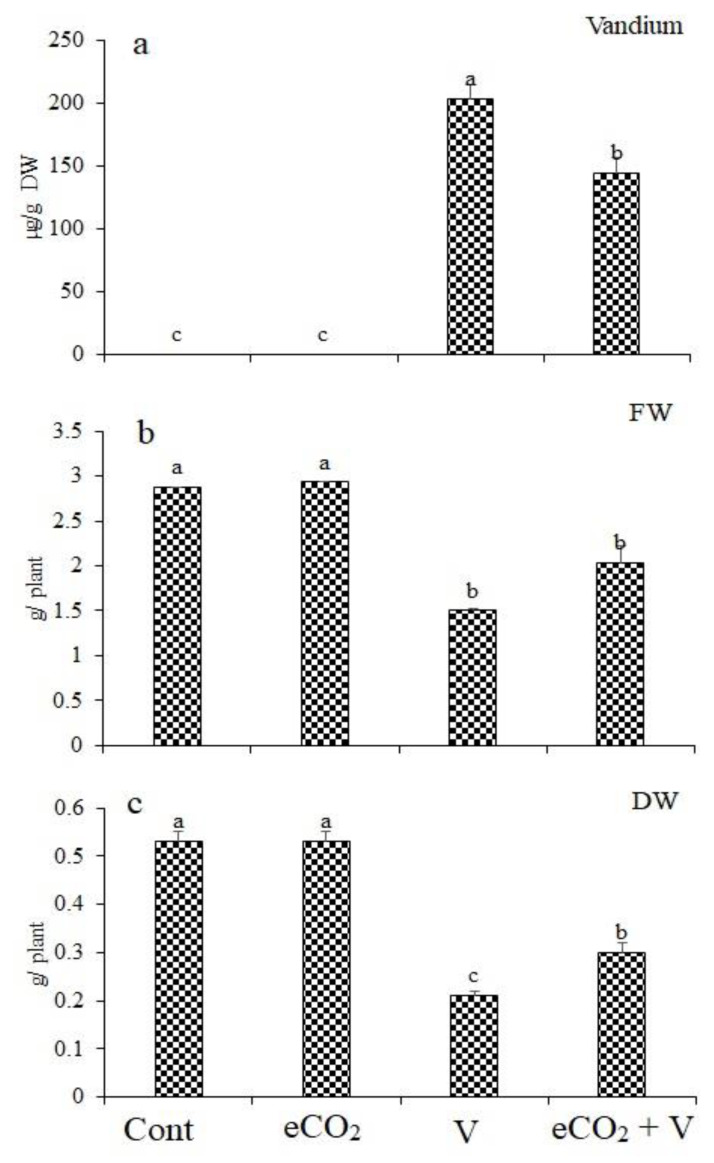
Effect of elevated CO_2_, vanadium (V) and their combination on (**a**) vanadium concentration, (**b**) fresh weight (FW) and (**c**) dry weight (DW) of wheat. Cont.: ambient CO_2_ (410 ppm); eCO_2_: (620 ppm), V:350 mg/kg soil. The aforementioned information is presented as mean values with standard error (*n* = 4). One-way ANOVA and the Tukey posthoc test were used to statistically examine the data and compare the means. Different letters denote statistically significant differences between the means of the same plant species, at least at the 0.05 level of significance.

Wheat plants exposed to V stress accumulated notable amounts of V in their shoots (Figure 1). A remarkable reduction in V accumulation (29% reduction) can be observed in Vanadium (Na_3_VO_3_/Kg Soil) -stressed plants grown under eCO_2_ compared to those (Na_3_VO_3_/Kg Soil)-stressed plants grown without eCO_2_ (Figure 1a). The accumulation of V in wheat tissues resulted in significantly reduced fresh weight by 47.7% (Figure 1b). This reduction was abolished upon treatment with eCO_2_. Treatment with eCO_2_ alone had no significant effect on fresh or dry weights. In contrast, V treatment significantly reduced dry weight by 60% (Figure 1c). eCO_2_ reduced the effect of V on dry weight, where the decrease was only 29.2%.

### 2.2. Photosynthesis, Gas Exchange and Pigments

The effects of eCO_2_, V and their combination on pigments content, photosynthesis, and gas exchange in wheat were estimated (Table 1). Both chlorophyll a (Chl a) and chlorophyll b (Chl *b*) were reduced significantly in wheat upon V stress by 65% and 50%, respectively. This reduction, however, was significantly attenuated by eCO_2_ in Chl *a*, while a significantly increase in Chl *b* was observed. When wheat was treated with eCO_2_, the amount of carotenoids did not change significantly (*p* ˂ 0.05). However, when wheat was treated with vanadium or both vanadium and eCO_2_, the amount of carotenoids increased by 180% and 323%, respectively.

The changes in chlorophyll content were reflected as a significantly reduction in photosynthetic rate in V stressed plants by 47.7% and as a significantly increase by 19.11% under eCO_2_ conditions. Gas exchange in leaves was not significantly affected by all treatments. RuBisCO significantly decreased under both V alone and the combination of V and eCO_2_ by 66.2% and 42.2%, respectively, on the other hand, eCO_2_ caused non-significant increase.

### 2.3. Organic Acids and Phenolic Conten in Soil

As they are known as nature chelating agents that play a role in reducing heavy metal uptake, oxalic acid and citric acid were measured. The effect of different treatments on oxalic acid, citric acid, and phenol contents in soil showed a significant increase under all three treatments (Table 2). This increase showed the higher amounts under the combined eCO_2_ and V treatments by 93%, 471%, and 228%, respectively.

### 2.4. Quantification of Oxidative Markers

**Figure 2:**
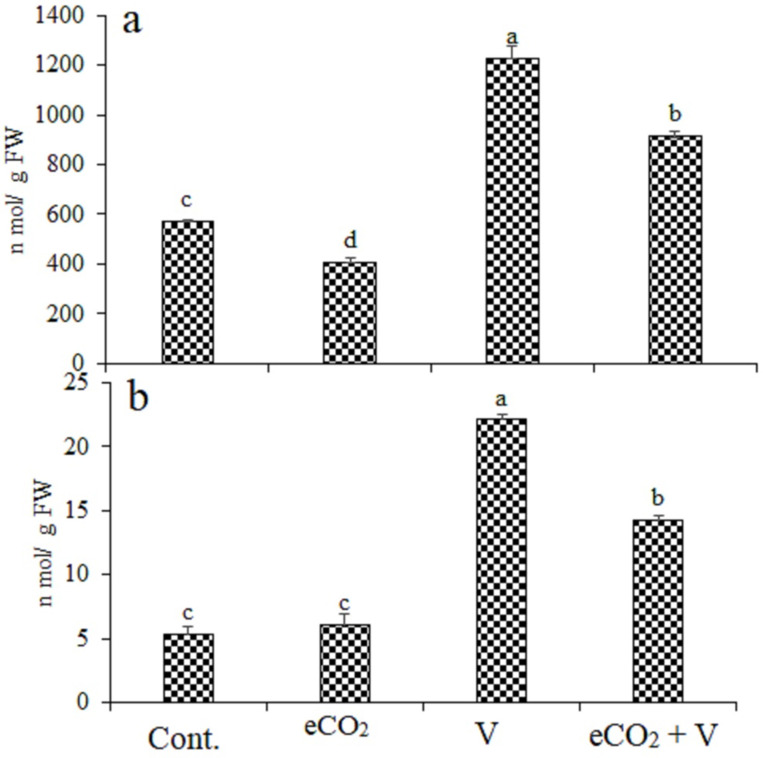
Effect of elevated CO_2_, vanadium (V) and their combination on (**a**) hydrogen peroxide (H_2_O_2_) and (**b**)) malondialdehyde (MDA) of wheat. Cont.: ambient CO_2_ (410 ppm); eCO_2_: (620 ppm), V:350 mg/kg soil. The aforementioned information is presented as mean values with standard error (*n* = 4). One-way ANOVA and the Tukey post hoc test were used to statistically examine the data and compare the means. Different letters denote statistically significant differences between the means of the same plant species, at least at the 0.05 level of significance.

Treatments with V, eCO_2_, and their combination induced oxidative stress in wheat shoots compared with their control plants, as indicated by the increased content of H_2_O_2_ and MDA. (Figure 2). Vanadium treatment caused an increase by 114% and 317% in H_2_O_2_ and MDA, respectively. V Treatment increased H_2_O_2_ and MDA by 114.8% and 317%, respectively, while these increases were reduced by eCO_2_ (59.7% and 167.9%, respectively), indicating less oxidative damage in V treated plants under eCO_2_. Treatment by eCO_2_ alone cause a significantly decrease in H_2_O_2_ by 28% and non-significant increase in MDA by 15%.

### 2.5. Nonenzymatic Antioxidants

TAC activity exhibited significant increase under V and combination of V and eCO_2_ treatments by 64.8% and 120%, respectively (Table 3), while eCO_2_ caused a non-significant increase. Significant increase in phenolics content could be observed of plants exposed to V or V+eCO_2_ compared to their control values, by 219% and 293%, respectively (Table 3). Moreover, a pronounced increase in flavonoids and could be observed in all three treatments, where the combined treatment of V and elevated CO_2_ was the most significant (by more than five times). Table 3 shows the protective role of tocopherols against V-induced stress in wheat. eCO_2_ significantly increased each of the alpha, beta, and sigma tocopherols by 43.3%, 338%, and 20.6%, respectively. Similarly, these tocopherols were increased by V treatment by 146.6%, 315.3%, and 66.1%, respectively (Table 3). The combination effect of V and CO_2_ exhibited significant increase by 326%, 500%, and 171% for the alpha, beta, and sigma, respectively (Table 3).

### 2.6. Antioxidant Enzymes

Changes in the activity of the direct POX, SOD, and CAT were recorded. Under eCO_2_ stress, APX, ASC, GSH, and SOD enzyme activities showed non-significant increases in their activities compared to their control values (Table 4), but significant increases were recorded in DHAR, GR, GPX, POX, and CAT enzyme activities by 176%, 49.1%, 81.3%, 27%, and 78%, respectively. The treatment of V only caused significant increases in GSH (238%), ASC (104.4%), DHAR (780%), GR (322%), GPX (500%), POX (540%), CAT (372%), SOD (35.3%), and APX (135%). The previous increases were less than those caused by the combination of vanadium and elevated carbon for the most studied enzymes. The treatment of V+eCO_2_ were as follow: GSH (290%), ASC (197%), GR (597%), GPX (663%), SOD (105%), and APX (242%), indicating that the treatment that of the combination of V and eCO_2_ showed the biggest increase except CAT enzyme (Table 4).

### 2.7. Heavy Metals Chelating Proteins

Table 5 shows the changes in metallothioneins (MTC), phytochelatins (PC), Tgsh, and GST activity in wheat plants exposed to eCO_2_, V, or their combination. MTC, GST and PC contents in wheat were enhanced when plants were exposed to V treatment by 8.4%, 168.4% and 106%, respectively. Under eCO_2_ treatment, MTC and were non-significantly decreased relative to their controls. The combination of V and eCO_2_ treatment improved the wheat shoots content of GST activity, the content of MTC, PC and GST Activity by 158%, 279% and 284%, respectively (Table 5).

## 3. Discussion

Even if there is a growing demand for crops, soil V pollution will significantly cut down on the amount produced. It will be challenging to satisfy this demand. The impacts of heavy metal stress are mitigated by eCO_2_. Similar to earlier research [19,36], we also found that eCO_2_ did reduce the V toxicity on growth, physiology and oxidative status level. Although the protective effect of eCO_2_ against heavy metal stress is well studied [36], the physiological and biochemical bases underlying the V stress mitigating impact of eCO_2_ is not studied. Here, we show that eCO_2_ effectively reduced V toxicity in wheat, proving that eCO_2_ is a useful and effective technique for lowering V accumulate and toxicity. This result is consistent with a recent investigation on rice plants, in which the presence of carbon dioxide led to an increase in chromium uptake and toxicity in plants.

V-induced V accumulation in wheat plants can explain the growth decrease. Plants can absorb V in place of phosphorus [32] and consequentially limits nutrients uptake and inhibit plant growth. The findings imply that V accumulation led to a reduction in biomass accumulation. This might be attributed, at least in part, to the decline in photosynthetic activity and Chl *a* and Chl *b* contents. Similar to this, rice seedlings were vulnerable to V that was correlated with decreased photosynthesis [37]. V significantly reduced the manufacture of Chl and interfered with amino acids that included sulfur, which reduced plant metabolism [38]. Our findings are supported by the observation that rice seedlings exposed to increasing V concentrations exhibit a concentration-dependent decrease in Chl *a* and *b* content and photosynthetic rate [37]. Additionally, Olness and Palmquist [39] demonstrated that V hampered soybean roots’ ability to absorb calcium and magnesium.

Here, wheat has a decreased fresh weight that eCO_2_ helps to somewhat offset. V has also been demonstrated to decrease pigment buildup in a number of plant species [40,41]. This supported our findings since plants have lower levels of chlorophyll *a* and *b* and lower levels of photosynthetic activity. Wheat responded more strongly to eCO_2_ levels, with reduced V absorption and higher fresh weight being observed. According to Krupa and Baszynski [42] and Zeid [43], V has an impact on the dark-and-light interaction, which is supported by our reduced observations of photosynthesis. It modifies the enzymatic processes involved in photosynthetic carbon fixation and plastid structure [44]. To reduce V buildup in plant tissue, eCO_2_ reduced stomatal conductance. Previous studies showed that gas exchange decreased under eCO_2_, according to reports [25,45]. According to our findings, wheat benefited more from eCO_2_, which encouraged plant development. The impact of eCO_2_ on heavy metal stress reduction has also been observed in various research studies [43,44]. Here, we demonstrate that V experiences the same benefits as eCO_2_ and that both rice cultivars do as well.

V interacts with several proteins and has an impact on a variety of biological processes, including lipid peroxidation and membrane-bound transport systems [46]. V treatment induced oxidative stress in wheat plants, as evidenced by increased MDA levels in the roots and decreased photosystem II quantum efficiency (lower Fv/Fm value). Similar to this, rice seedlings subjected to V stress displayed an increase in oxidative stress indicators such as H_2_O_2_, MDA, electrolyte leakage, and a decrease in photosystem II quantum efficiency [10]. H_2_O_2_ and MDA concentrations were also significantly enhanced in chickpea plants [38,47]. As a result, there was a positive increase in both cell death and ion leakage. V also stimulated the haloperoxidase gene in plant tissue [48], which led to serious oxidative damage. To reduce stress severity, several network genes, including peroxidases, MDHAR, GR, glutaredoxin, thioredoxin, and GST, were increased by V stress in rice according to Lin and Trinh’s study [49]. Plant tissues promote the manufacture of metal chelators such MTC and PC as well as the metal detoxifying enzyme GST to combat V toxicity [32,50]. According to Roychoudhury [32], V significantly increased the activity of antioxidative enzymes such as SOD, CAT, and POX, where CAT activity allows for the removal of H_2_O_2,_ and POX protects cellular membranes from oxidative damage. In line with our results, in chamomile plants, SOD was activated when exposed to V [51]. Additionally, when stressed with V, hydroponically produced wheat had increased APX and GR activity [16]. V stress caused an increase in CAT and APX activities in wheat, which may be a result of the oxidative stress that wheat is more likely to experience due to its type of photosynthesis.

Interestingly, eCO_2_ reduced V induced oxidative damage in wheat plants. This mitigation effect is previously reported in several studies [47,48,49]. Increased CO_2_ has the ability to provide additional C for antioxidant production. Importantly, eCO_2_ can maintain the C skeletons and the energy that stressed plants need to grow [52]. According to AbdElgawad et al. [34], increasing the availability of C under eCO_2_ lead to an increase in antioxidant molecules production, improving protection against oxidative damage. Wheat’s antioxidant enzymes were boosted by eCO_2_ settings under environmental stress [53]. As a result of the V exposure, APX, GPX, CAT, SOD, DHAR, and GR activities are increased, although to a greater extent in plants exposed to eCO_2_ levels. In addition to improved antioxidant production, eCO_2_ reduced ROS production and photorespiration [34]. In the current research, eCO_2_ significantly decreased increases in photorespiration, including HDR, GO, and G/S ratio, caused by Cr. Prior research by Zinta et al. [21] demonstrated that eCO_2_ enhanced carboxylation over oxygenation of RuBisCO enzymes, thereby decreasing the production of ROS.

Together, this sheds light on how eCO_2_ can mitigate to the phytotoxicity hazards of V in wheat plants. Our work shows that the beneficial effect of eCO_2_ in improving the negative impact of soil V was linked to their potentiality to improve plant photosynthesis, which in turn provided energy and carbon backbone for scavenging the V-stress induced ROS accumulation.

## 4. Materials and Methods

### 4.1. Plant Growth and Treatments

We collected wheat seeds from the Agricultural Research Center in Giza, Egypt (*Triticum aestivum* L., Sids 13). Surface sterilization was carried out using sodium hypochlorite (0.5% *v*/*v*; 20 min). After the grains grew in wet perlite, they were transplanted into pots (cm high and 15 cm in diameter) with natural soil (0.5 kg) with the following characters: sand: 70%, Caly 30%, pH: 7.2, Organic matter: 1.1%, Nitrogen: 25 (µg/g DW), P: 1.6 (µg/g DW), K 75(µg/g DW), Fe: 0.84 (µg/g DW), Mn: 1.3 (µg/g DW), Zn: 0.1 (µg/g DW), V: zero. 20. Throughout the growth period, 3 cm of surface standing water was applied to all pots equally. A base fertilizer containing 1.2 g of K_2_HPO_4_3H_2_O (Sigma, Germany, Taufkirchen) as a nutrient source and 1.2 g of urea (containing 46% nitrogen) was administered. Under carefully controlled conditions (12 h of photoperiod, 350 mol photons m-^2^ s-^1^, 80% humidity, and 28/24 °C Day/night temperatures), pots were moved into growth-controlled cabinets at Jeddah university. Before starting the experiment, soil was spiked with vanadium (V) (350 Na_3_VO_3_ mg/kg soil). Non-spiked soil served as the control treatments. Then, after sowing the wheat seeds, spiked and non-spiked soils were grown under two climate conditions (i.e., ambient CO_2_ (aCO_2_, 420 ± 13 ppm) and (2) elevated CO_2_ (eCO_2_, 720 ± 21 ppm)). Thus, the following circumstances were used to cultivate wheat plants: (1) aCO_2_ + non- spiked soil (control); (2) eCO_2_ + non- spiked soil; (3) aCO_2_ + V spiked soil; and (4) eCO_2_ + V spiked soil. Following the completion of a preliminary experiment with a range of V concentrations (50–500 mg/kg soil), the most effective V concentration was chosen to reduce the growth (DW) of the delicate wheat without killing the plants [13]. All pots were watered daily to maintain a soil water content (SWC) of 78% throughout the experiments. Pots were weighted up daily to compensate the loss of water (25 to 40 mL/day) throughout all plants. After five weeks of growth, samples (shoots) were harvested and stored at 80 °C for further biochemical analysis. Additionally, soil samples were gathered for chemical examinations.

### 4.2. Organic Acids and Phenolic Content in Soil Samples

The dug roots were gradually agitated to isolate them from the bulk soils in order to acquire the rhizosphere. The levels citric acid, oxalic acid, and phenolic content were measured [54,55]. Using ribitol as an internal standard, organic acids (citric and oxalic acids) were collected in 0.1% phosphoric acid that also included butylated hydroxyanisole. As stated by de Sousa et al. [54], filtrates were employed for HPLC quantification using a LaChrom L-7455 diode array (Merck-Hitachi, Barcelona, Spain) after centrifugation. According to Zhang et al. [56], the phenolic content was spectrophotometrically estimated (Shimadzu UV-Vis 1601 PC, Kyoto, Japan).

### 4.3. Quantification of Total Vanadium

A known volume of an 8:1:1 solution of HNO_3_, H_2_SO_4_, and HClO_4_ was digested at 120–130 °C for 5 h using a known weight of dried plant material [57]. The clear aliquot was cooled, then diluted with deionized water to 25 mL for the V analysis. A 0.1 g soil fraction was heated until totally dissolved in a solution of concentrated HF, HNO_3_, and HClO_4_ (in a ratio of 12:1:2 (*v*/*v*)). A Perkin-Elmer spectrophotometer with an Atomic Absorption Model 800 was used for all measurements (Perkin-Elmer, Shelton, USA). Standard solutions of 10, 20, 40, 80, 160, and 200 g V L^−1^ in the matching sample matrix were made using a standard stock solution containing 1 g L^−1^ V (All chemical were Sigma, Germany, Taufkirchen).

### 4.4. Quantification of Photosynthetic Related Parameters

Prior to sample collection, treated wheat leaves had their stomatal conductance and light-saturated photosynthetic rate evaluated using a LI-COR LI-6400 (LI-COR Inc., Lincoln, NE, USA) [58]. The photochemical efficiency (Fv/Fm, photosystem ll system efficiency) was measured with a fluorimeter (PAM2000, Walz, Germany). Hemphill and Venketeswaran [58] state that after the shoots were homogenized in acetone, the concentrations of chlorophyll a and b, as well as carotenoids, were determined in the supernatant. Ribulose-1,5-bisphosphate carboxylase/oxygenase (RuBisCO) activities were investigated by Sulpice et al. [59]. The activity was measured in HEPES/KOH (pH 7.5, 50 mM) reaction buffer (glycerol (20%), BTriton-X100 (1%), SA (0.25%), EGTA (1 mM), MgCl_2_ (10 mM), benzamidine (1 mM), EDTA (1 mM), e-aminocapronic acid (1 mM), mM PMSF (1 mM), DTT (1 mM), and leupeptin (10 mM), all chemicals were from Sigma, Germany.

### 4.5. Quantification of Oxidative Damage Markers

By observing the peroxide-mediated oxidation of Fe^2+^ and the subsequent reaction of Fe^3+^ with xylenol orange using the FOX1 technique, the level of H_2_O_2_ was determined [60]. The Fe^3+^ xylenol orange complex’s absorbance was measured at 560 nm, and the reaction mixture with catalase was used to test its reaction specificity for H_2_O_2_. Wheat tissues were homogenized in 80% ethanol to extract the lipid peroxidation level, which was then measured using the TBA-MDA reagent [61]. According to Steczko et al. [62], the activity of lipoxygenase (LOX) was determined by extracting it in 50 mM potassium phosphate buffer (pH 7.0), 10% polyvinyl pyrrolidone (PVP), 0.25% triton X-100, and 1 mM polymethyl sulfonyl fluoride (PMSF) at 560 nm, and the reaction mixture with catalase was used to test its reaction specificity for H_2_O_2_ (CAT). By monitoring changes in conjugated dienes, the activity of LOX was computed and measured. Oxidative maerakers analyses measured spectrophotometry by using a microplate reader microplate reader (Synergy Mx; BioTek Instruments Inc., Vermont, VT, USA).

### 4.6. Quantification of Antioxidant Parameters

Antioxidant concentrations and total antioxidant capacity were extracted using 80% ethanol and centrifugation at 14,000× *g* for 18 min at 4° C. Using a Trolox standard solution (0–650 M) and the “Ferric Reducing Antioxidant Power” assay (FRAP reagent, 0.3 M acetate buffer (pH 3.6), 0.01 mM TPTZ in 0.04 mM HCl, and 0.02 M FeCl3.6H_2_O) [55]. By using HPLC (Shimadzu, Hertogenbosch, The Netherlands) analysis, ascorbate (AsA) and glutathione (GSH) were identified. 6% (w/v) meta-phosphoric acid was used to remove frozen plant tissue, and a reversed-phase HPLC column (100 4.6 mm Polaris C18-A, 3 m particle size, 40 °C) was used to separate the antioxidants [30]. In 80% ethanol (*v*/*v*), polyphenols and flavonoids were extracted (MagNALyser, Belgium). Total phenolic and flavonoid content were assessed using the Folin-Ciocalteu and aluminum chloride assays, respectively [63,64]. Proteins were extracted to determine the activity of antioxidant enzymes in two mL of a KPO_4_ extraction buffer containing polyvinylpyrrolidone (10% *w*/*v*), Triton X-100 (0.25% *v*/*v*), and phenylmethylsulfonyl fluoride at a pH of 7.0 (PMSF, 1 mM). 0.05 M MES/KOH was used to spectrophotometrically assess the activities of dehydr-ASC reductase (DHAR, EC 1.8.5.1), GSH reductase (GR, EC 1.6.4.2), ascorbate peroxidase (APX), and monodehydro-ASC reductase (MDHAR, EC 1.6.5.4). The oxidation of pyrogallol [65] and the suppression of NBT reduction at 560 nm were used to measure the activities of peroxidase (POX, EC 1.11.1.6) and superoxide dismutase (SOD, EC 1.15.1.1) enzymes, respectively. The rates of H_2_O_2_ oxidation at 240 nm [66] and NADPH reduction at 340 nm [67] were used to measure the activities of catalase (CAT, EC 1.11.1.6) and glutathione peroxidase (GPX, EC 1.11.1.9). Enzyme activity was adjusted to the total soluble protein content using the Lowry method [68]. All metabolites and enzymes analyses were scaled down for semi–high-throughput analysis using a microplate reader microplate reader (Synergy Mx; BioTek Instruments Inc., Vermont, VT, USA).

### 4.7. Quantification of Detoxification Related Parameters

A KPO_4_ buffer (50 mM, pH 7.0) containing 0.5 mM CDNB and 1 mM GSH was used to extract glutathione-S-transferase (GST; EC 2.5.1.18). The activity was valued in accordance with [69]. In accordance with Diopan et al. [70], the concentration of metallothionein (MTC) was electrochemically determined using differential pulse voltammetry Brdicka reaction. After being extracted with 5% sulfosalicylic acid and combined with Ellman’s reagent, total phytochelatins (total thiols-non-protein) were determined by spectrophotometry at 412 nm [71].

### 4.8. Statistical Analysis Experiments

Four duplicates of each treatment (*n* = 4) were used in the experiments, which used a fully randomized block design according to de Sousa et al. [38], SPSS (Chicago, IL, USA) and R (Team, R.C., 2013). Levene’s and the Kolmogorov-Smirnov (SPSS)/Shapiro-Wilk (R) tests were employed to assess the homoscedasticity and normality of the data, respectively. Two-way and four-way analysis of variance (ANOVA) was performed on all data (*p* ˂ 0.05). For further pairwise statistical comparison of means, a Duncan’s (by SPSS), following two-way ANOVA, and/or Tukey HSD test (R), following four-way ANOVA, were used.

## 5. Conclusions

This study advances our knowledge of the processes behind the variations in the physiological and biochemical responses of the wheat crop under V and eCO_2_ conditions. Despite the fact that plants react to stress in largely identical ways, cultivar-specific variations might nonetheless exist. According to this study, eCO_2_ reduces the effects of V stress by elevating antioxidant levels and the activity of antioxidant enzymes. However, when V is present, wheat is able to detoxify it and absorb less of it under future CO_2_ levels, perhaps by upregulating transporters and boosting phytochelat levels.

## Figures and Tables

**Table 1:** Effect of elevated CO_2_ (eCO_2_), vanadium (V) and their combination on (A) chl *a* (mg/gFW), (B) Chl *b* (mg/g FW), (C) carotenoids (mg/g FW) (D), photosynthesis (Asat; mol CO_2_/m^2^ S), (E) stomatal conductance (gs; nmol CO_2_ m^−2^ s ^−1^), and (F) RuBisCO (nmol 3-PGA/mg protein. min) of wheat. Data are mean values ± SE (*n* = 4). Using Fisher’s LSD test, the data for each group were compared pairwise (*p* < 0.05; *n* = 4). Different letters indicate that there is a significant difference between the treatments.

	Photosynthesis μmol m^–2^ sec^–1^ (mol CO_2_/m^2^S)	gs	RuBisCO (nmol 3-PGA/mg Protein-min)	Chl *a* (mg/g FW)	Chl *b* (mg/g FW)	Carotenoids (mg/g FW)
Control (ambient CO_2_—410 ppm)	13.50 ± 0.01 ^b^	0.62 ± 0.0 ^a^	38.80 ± 3.0 ^a^	2.80 ± 0.2 ^a^	0.18 ± 0.0 ^b^	0.21 ± 0.0 ^c^
eCO_2_ (620 ppm)	16.08 ± 0.9 ^a^	0.52 ± 0.1 ^a^	41.78 ± 2.0 ^a^	2.50 ± 0.1 ^a^	0.27 ± 0.0 ^a^	0.22 ± 0 ^c^
V (350 mg/kg)	7.06 ± 0.4 ^d^	0.60 ± 0.01 ^a^	13.10 ± 0.2 ^c^	0.98 ± 0.1 ^c^	0.09 ± 0.0 ^c^	0.59 ± 0 ^b^
eCO_2_ + V	11.08 ± 0.1 ^c^	0.64 ± 0.0 ^a^	22.10 ± 4.0 ^b^	2.10 ± 0.1 ^b^	0.15 ± 0.0 ^ab^	0.89 ± 0.1 ^a^

**Table 2:** Effect of elevated CO_2_ (eCO_2_), vanadium (V) and their combination (eCO_2_ + V) on vanadium, citric acid and oxalic acid in soil. Data are mean values ± SE (*n* = 4). Using Fisher’s LSD test, the data for each group were compared pairwise (*p* < 0.05; *n* = 4). Different letters indicate that there is a significant difference between the treatments.

	Microg Vanadium	Phenol (µg/gm)	Citric Acid (µg/gm)	Oxalic Acid (µg/gm)
Control (ambient CO_2_—410 ppm)	0 ± 0.0 ^c^	21.5 ± 2.0 ^ac^	1.07 ± 0.06 ^c^	4.9 ± 0.6 ^d^
eCO_2_ (620 ppm)	0 ± 0.0 ^c^	55.3 ± 1.0 ^b^	3.26 ± 0.14 ^b^	6.1 ± 0.3 ^c^
V (350 mg/kg)	104.9 ± 4.0 ^b^	59.4 ± 2.0 ^b^	4.50 ± 0.08 ^b^	7.3 ± 0.3 ^b^
eCO_2_ + V	187.0 ± 5.0 ^a^	70.7 ± 3.0 ^a^	6.12 ± 0.30 ^a^	9.5 ± 0.6 ^a^

**Table 3:** Effect of elevated CO_2_ (eCO_2_), vanadium (V) and their combination (eCO_2_ + V) on non-enzymatic antioxidant of wheat. total antioxidant capacity (TAC), Polyphenol (Pphenol), flavonoid (Flav) and. Data are mean values ± SE (*n* = 4). Using Fisher’s LSD test, the data for each group were compared pairwise (*p* < 0.05; *n* = 4). Different letters indicate that there is a significant difference between the treatments. Data are mean values ± SE (*n* = 4). Using Fisher’s LSD test, the data for each group were compared pairwise (*p* < 0.05; *n* = 4). Different letters indicate that there is a significant difference between the treatments.

	TAC	Pphenol	Flav	alpha Toco	beta Toco	Sigma Toco
	mg/gFW					
Control (ambient CO_2_—410 ppm)	29.3 ± 1.0 ^c^	1.22 ± 0.40 ^b^	0.56 ± 0.0 ^d^	3.0 ± 0.70 ^d^	1.3 ± 0.43 ^c^	21.3 ± 0.43 ^c^
eCO_2_ (620 ppm)	31.1 ± 3.0 ^c^	1.34 ± 0.20 ^b^	0.94 ± 0.10 ^c^	4.3 ± 0.90 ^c^	5.7 ± 0.92 ^b^	25.7 ± 0.92 ^b^
V (350 mg/kg)	48.3 ± 8.0 ^b^	3.90 ± 0.30 ^a^	2.28 ± 0.60 ^b^	7.4 ± 2.00 ^b^	5.4 ± 0.96 ^b^	35.4 ± 0.96 ^d^
eCO_2_ + V	64.5 ± 2.0 ^a^	4.80 ± 0.40 ^a^	3.62 ± 0.20 ^a^	12.8 ± 4.00 ^a^	7.8 ± 1.34 ^a^	57.8 ± 1.34 ^a^

**Table 4:** Effect of elevated CO_2_ (eCO_2_), vanadium (V) and their combination (eCO_2_ + V) on antioxidant parameters of wheat. Reduced glutathione (GSH), ascorbate (ASC), dehydroascorbate reductase (DHAR), glutathione reductase (GR), peroxidase (GPX), peroxidase (POX), peroxidase (POX), catalase (CAT), superoxide dismutase (SOD) and ascorbate peroxid (APX). Data are mean values ± SE (*n* = 4). Using Fisher’s LSD test, the data for each group were compared pairwise (*p* < 0.05; *n* = 4). Different letters indicate that there is a significant difference between the treatments.

	GSH	ASC	DHAR	GR	GPX	POX	CAT	SOD	APX
	mg/gFW		nmo/min.mg Protein						
Control (ambient CO_2_—410 ppm)	0.031 ± 0 ^c^	0.089 ± 0.0 ^c^	0.021 ± 0.0 ^c^	0.122 ± 0.01 ^d^	0.102 ± 0.012 ^d^	0.506 ± 0.06 ^c^	2.73 ± 0.1 ^d^	150 ± 2 ^d^	0.14 ± 0.01 ^c^
eCO_2_ (620 ppm)	0.027 ± 0.0 ^c^	0.105 ± 0.0 ^c^	0.058 ± 0.01 ^b^	0.182 ± 0.01 ^c^	0.185 ± 0.01 ^c^	0.643 ± 0.10 ^b^	4.86 ± 0.24 ^c^	148 ± 11.0 ^d^	0.16 ± 0.01 ^c^
V (350 mg/kg)	0.105 ± 0.0 ^b^	0.182 ± 0.0 ^b^	0.185 ± 0.0 ^a^	0.515 ± 0.02 ^b^	0.612 ± 0.0 ^b^	3.24 ± 0.06 ^a^	12.9 ± 0.13 ^a^	203 ± 07 ^b^	0.33 ± 0.04 ^b^
eCO_2_ + V	0.121 ± 0.0 ^a^	0.265 ± 0.0 ^a^	0.178 ± 0.04 ^a^	0.851 ± 0.0 ^a^	0.779 ± 0.08 ^a^	3.149 ± 0.50 ^a^	8.3 ± 0.16 ^b^	304 ± 10.0 ^a^	0.48 ± 0.02 ^a^

**Table 5:** Effect of elevated CO_2_ (eCO_2_), vanadium (V) and their combination (eCO_2_ + V) on reduced glutathione (GSH), total glutathione (Tgsh), Metallothioneins (MTC) and glutathione-S-transferase (GST). Data are mean values ± SE (*n* = 4). Using Fisher’s LSD test, the data for each group were compared pairwise (*p* < 0.05; *n* = 4). Different letters indicate that there is a significant difference between the treatments.

	Phytochelatins	Tgsh (nmol/gFW)	MTC (nmol/gFW)	GST (nmol/min.mg Protein)
Control (ambient CO_2_—410 ppm)	3.98 ± 1.0 ^c^	0.120 ± 0.0 ^c^	29.2 ± 3.0 ^c^	0.19 ± 0.06 ^c^
eCO_2_ (620 ppm)	3.50 ± 0.14 ^c^	0.160 ± 0.14 ^c^	24.1 ± 0.9 ^c^	0.13 ± 0.14 ^c^
V (350 mg/kg)	8.20 ± 0.80 ^b^	0.840 ± 0.08 ^b^	52.1 ± 1.0 ^b^	0.51 ± 0.08 ^b^
eCO_2_ + V	15.10 ± 0.60 ^a^	0.839 ± 0.20 ^a^	75.4 ± 6.0 ^a^	0.73 ± 0.06 ^a^

## Data Availability

The data presented in this study are available on request from the corresponding author.
